# CVD Grown CNTs-Modified Electrodes for Vanadium Redox Flow Batteries

**DOI:** 10.3390/ma17133232

**Published:** 2024-07-01

**Authors:** Yi-Sin Chou, Nitika Devi, Yan-Ting Lin, Amornchai Arpornwichanop, Yong-Song Chen

**Affiliations:** 1 Department of Mechanical and Systems Engineering, National Atomic Research Institute, 1000 Wenhua Rd., Taoyuan 325207, Longtan District, Taiwan; yschou@nari.org.tw (Y.-S.C.); yantinglin@nari.org.tw (Y.-T.L.); 2 Advanced Institute of Manufacturing with High-Tech Innovations and Department of Mechanical Engineering, National Chung Cheng University, 168 University Rd., Minhsiung Township 621301, Chiayi County, Taiwan; nitika19967@gmail.com; 3 Center of Excellence in Process and Energy Systems Engineering, Department of Chemical Engineering, Faculty of Engineering, Chulalongkorn University, 254 Phayathai Rd., Bangkok 10330, Thailand; amornchai.a@chula.ac.th

**Keywords:** vanadium redox flow battery, chemical vapor deposition, carbon nanotube, energy efficiency

## Abstract

Vanadium redox flow batteries (VRFBs) are of considerable importance in large-scale energy storage systems due to their high efficiency, long cycle life and easy scalability. In this work, chemical vapor deposition (CVD) grown carbon nanotubes (CNTs)-modified electrodes and Nafion 117 membrane are utilised for formulating a vanadium redox flow battery (VRFB). In a CVD chamber, the growth of CNTs is carried out on an acid-treated graphite felt surface. Cyclic voltammetry of CNT-modified electrode and acid-treated electrode revealed that CNTs presence improve the reaction kinetics of V^3+^/V^2+^ and VO_2_^+^/VO^2+^ redox pairs. Battery performance is recorded for analysing, the effect of modified electrodes, varying electrolyte flow rates, varying current densities and effect of removing the current collector plates. CNTs presence enhance the battery performance and offered 96.30% of Coulombic efficiency, 79.33% of voltage efficiency and 76.39% of energy efficiency. In comparison with pristine electrodes, a battery consisting CNTs grown electrodes shows a 14% and 15% increase in voltage efficiency and energy efficiency, respectively. Battery configured without current collector plates performs better as compared to with current collector plates which is possibly due to decrease in battery resistance.

## 1. Introduction

Energy scarcity is threatening the world, so it is necessary to shift toward more sustainable energies that rely on renewable energy resources [1]. Renewable energy wide utilisation is limited by its intermittence nature which can be alleviated by building up efficient energy storage devices. Energy storage devices like supercapacitors, batteries, fuel cells, and redox flow batteries (RFBs) etc., can store energy from renewable energy resources and can also work independently [2,3]. RFBs can play a crucial role in sustainable energy storage due to long cycle life, high energy efficiency, easy scalability and high safety [4].

Scientists have developed different types of RFBs such as all-vanadium RFBs (VRFBs), all-iron RFBs (IRFBs), Fe-Cr RFBs, Zn-Br_2_ RFBs etc., [5]. Out of all kinds, VRFBs are the most explored and advanced redox flow battery (RFB) due to their high stability and high energy efficiency. Large-scale VRFBs can deliver a capacity of few100 KWh to several MWh [6]. A VRFB stores energy through the reactions of V^3+^/V^2+^ and VO^2+^/VO_2_^+^ redox pairs in negative and positive electrolyte, respectively. Possibility of cross-contamination can be avoided in VRFBs as both negative and positive electrolytes utilise the vanadium ions in different oxidation states. A battery consists of various parts like a membrane, electrodes, bipolar plates, current collector plates, cell frame etc. Each part influences the battery performance and operation stability [7].

Electrodes are a crucial part of battery assembly because redox reactions kinetics are highly dependent on the conductivity and surface reactivity of the electrodes. Unlike conventional batteries, electrodes do not participate in chemical reactions but only provide an active surface area for chemical reactions of the electrolyte ions. Various forms of carbon, like graphite felt, carbon paper, carbon cloth etc., have been utilised for the use of VRFB electrodes. These cost-efficient and chemically stable electrodes have very low conductivity which impacts the battery performance [8]. Surface modification, composite formation, and doping etc., efforts have been employed to enhance the conductivity of electrodes. Jialin et al. [9] modified graphite felt electrodes with co-doping of nitrogen and phosphorous which can work for VRFBs at a high current density of 300 mA cm^−2^. Performance comparison with pristine electrode and heat-treated electrode, modified electrode showed 11% and 2% higher voltage efficiency at 300 mA cm^−2^, respectively. Liu et al. reported high-performance quaternary ammonium modified electrodes which can perform at a high current density of 300 mA cm^−2^ with a 60% energy efficiency and battery can operate for 1000 charge-discharge cycles at 200 mA cm^−2^ [10]. Surface properties like hydrophobicity and active surface area of the electrodes were modified with low-temperature atmospheric pressure plasma treatment. Modified electrodes exhibited higher energy efficiency and capacity of 77.6% and 2.08 Ah, respectively which was 67.9% and 1.47 Ah in pristine electrodes [11]. Highly conducting and high surface area carbon materials can also be utilised for the modification of electrodes which results in high performance of the battery [12]. It has been reported that both single-wall carbon nanotubes (SWCNTs) and multiwall carbon nanotubes (MWCNTs) [13] can be used as electrocatalysts for electrode modification [14]. Carbon nanotubes (CNTs)-modified electrodes prepared by surface coating method showed 80% energy efficiency at 200 mA cm^−2^ which was 15% higher than the untreated electrodes [15]. Gonzalez et al. [16] proposed a graphene modified electrode by electrophoretic deposition of graphite oxide solution and resulting electrodes were used with a combination of Nafion 117 membrane in VRFB. Battery remarkably improved the kinetics of VO^2+^/VO_2_^+^ pair and offered 95.8% energy efficiency at 25 mA cm^−2^. Layer-by-layer deposition of polyethylenimine and carbon nanotubes dispersed with poly(acrylic acid) was done on graphite felt. These modified electrodes showed less charge and discharge overpotential thus increasing the battery energy efficiency which was 7% higher than the energy efficiency of unloaded electrodes [17]. Thus, conducting and high surface area carbon materials can be successfully employed for electrode modifications. Electrode compression ratio also considerably affects the performance of VRFBs as reported that with increasing electrode compression, energy efficiency of VRFBs also increased [18]. Bipolar plates structure and design are also important in obtaining the optimum performance of VRFBs [19].

Membrane is an important part of the battery assembly which provides a path for proton transfer and hinders the mixing of positive and negative electrolytes. Battery performance is significantly degraded due to vanadium ions permeation because it leads to self-discharge and mixed potential production. Also, chemical stability of the membrane is a concerning factor as it should be sustained in harsh chemical conditions. Different types of membranes like sulfonated poly(ether ether ketone) (SPEEK), sulfonated poly(ether sulfone) (SPES), sulfonated poly(imide) (SPI) and polybenzimidazole (PBI) have been utilised in VRFBs [20]. But Nafion membrane is reported to have the best performance for VRFBs due to its chemical stability and high proton conductivity. In addition to type of membrane, battery performance is affected by the thickness and type of Nafion membrane. Coulombic efficiency is charge efficiency so it is governed by a membrane, and ohmic losses contribute to defining the voltage efficiency and energy efficiency. Jiang et al. [21] evaluated the battery performance with Nafion 112, Nafion 1135, Nafion 115, and Nafion 117 in a broad range of current density of 40–320 mA cm^−2^. Nafion 115 membrane have the highest energy efficiency and thicker Nafion 115 and Nafion 117 membranes are preferable choices for commercial VRFBs applications due to least electrolyte imbalance [21]. Another work compared the VRFB performance with Nafion 212 and Nafion 117 and found that Nafion 117 offered better battery performance as compared to Nafion 212. This was due to a dominant increase in Coulombic efficiency in comparison to an increase in the ohmic resistance due to thickness of Nafion 117 membrane [22]. Therefore, it is important to have a thicker membrane for stable battery performance.

This work demonstrated the effect of electrode modification, employing Nafion 117 membrane and role of current collector plates in battery performance. Here, we formulated a battery which consists of chemical vapour deposition (CVD)-grown CNTs-modified electrodes with a thick Nafion 117 membrane. Goal of present work was to evaluate best battery performance with a thick membrane and a modified electrode under harsh conditions like high electrolyte flow rate, high current densities and studied the effect of configurational changes. These findings will help in understanding that high electrolyte flow can be maintained with Nafion 117 without causing electrolyte cross-over. A battery configuration without current collector plates was developed and studied to see its effect on the resistance of battery. Prior to CVD treatment, PAN-based graphite felt electrodes underwent acid treatment. Battery performance was compared with a battery consisting of pristine electrodes. Important parameters like electrolyte flow rates and varying current densities were analysed and acceptable electrolyte flow rates and current density were applied for battery operations.

## 2. Materials and Methods

### 2.1. Acid Heat Treatment

In the preparation of acid heat treated electrode, graphite felt was dipped in 240 mL of 8 M nitric acid (ACS reagent, 70%, Alfa Aesar, Ward Hill, MA, USA) and 80 mL of 2 M sulfuric acid (ACS reagent, 95.0–98.0%, Alfa Aesar, Ward Hill, MA, USA), followed by heating at 100 °C for 10 h. Resulting graphite felt was washed with deionized water (DI) and then heated in a high-temperature furnace at 450 °C for 10 h in the air.

### 2.2. CNTs Processing (CNTs Composite Electrode Manufacturing Process)

**Figure 1:**
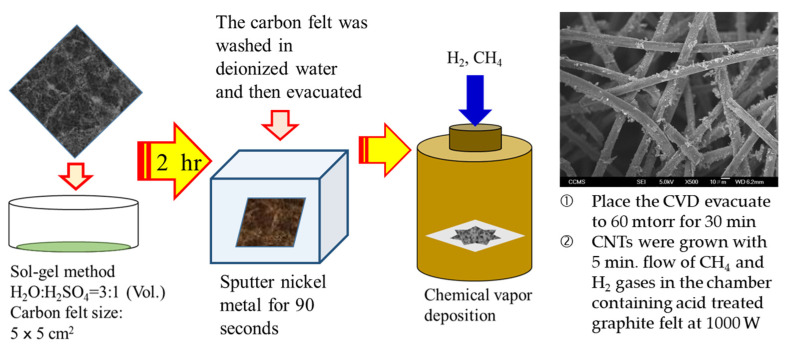
Schematic of CVD performed on acid-treated graphite felt.

CNTs were CVD-grown on graphite felt using CH_4_ as a precursor gas. Initially, graphite felt was treated with H_2_O:H_2_SO_4_ = 3:1 (Vol.) with heating at 100 °C for 2 h. Acid treated graphite felt was rinsed in DI water then filtered and vacuum treated. Acid treatment helped in changing the surface chemistry of graphite felt by making it less hydrophobic. Dried acid treated graphite felt was subjected to magnetron sputtering for 90 s, to deposit Ni. This step was followed by CVD, in a chamber with low pressure of 60 mtorr which was maintained for 30 min. CNTs were grown with 5 min. flow of CH_4_ and H_2_ gases in the chamber containing acid treated graphite felt at 1000 W. H_2_ was introduced to remove the impurities of graphite felt. A schematic of the discussed process is shown in Figure 1 and Field emission scanning electron microscopy (FESEM) image of CVD-grown CNTs-modified graphite felt.

### 2.3. Electrolyte, Cell Preparation and Experimental Conditions

Vanadium electrolyte was purchased (Hua Moly Industry (Plum-Monix Industry Co., Ltd., Changhua, Taiwan)) having analytical composition concentration: 1.87 MV, 5.62 MS, 0.263 M Si, 0.025 M Fe, 11,627 ppm total organic carbon (TOC).

Single cell was fabricated assembling electrodes, bipolar plates, current collector plates and Nafion 117 as a separator membrane. Area of the cell was 5 × 5 cm^2^. All the experiments were performed with a cut-off voltage of 0.7 V and protection voltage of 1.7 V at a current density of 40 mA cm^−2^ with an electrolyte flow rate of 50 mL min^−1^. To study the effect of electrolyte flow rates and varying current density, electrolyte flow rates and current densities were varied at 6, 12, 18, and 24 mL min^−1^ and 20, 40, 60, and 65 mA cm^−2^, respectively.

### 2.4. Physical Characterization

FESEM of CNTs-modified electrode was done using FE-SEM (JEOL JSM-6700F, JEOL, Tokyo, Japan) with an accelerating voltage of 5.0 kV. The magnification ratios for observing the appearance of CNT are 500, 10,000, and 50,000.

### 2.5. Electrochemical Measurement

Cyclic voltammetry (CV) was performed in a flat reaction tank (Flat Cell, EG&G PARC, Oak Ridge, TN, USA) with a potentiostat (CH Instruments, CHI 600E, Bee Cave, TX, USA) under an argon atmosphere at room temperature. CV measurements were done in vanadium electrolyte with three electrode electrochemical setup. Diameter of working electrode was 1 cm with 0.3 cm thickness and Ag/AgCl and platinum foil were used as reference and counter electrode, respectively. CV was performed for voltage window −0.7 to 1.2 V with a scanning rate of 5 mV S^−1^. Carbon/Teflon gas diffusion membrane was used as a leak proof for the entire battery so that it can prevent working electrode from being wetted and leaking.

## 3. Results and Discussions

### 3.1. Morphological Study

**Figure 2:**
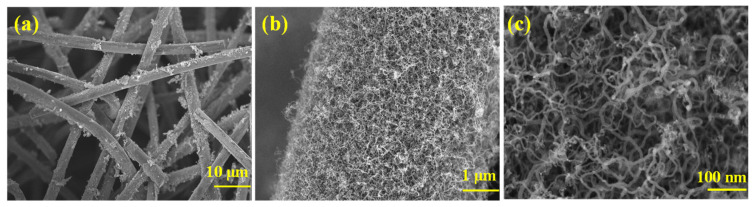
FESEM images of CNTs-modified electrode at different resolutions: (**a**) 10 μm; (**b**) 1 μm; (**c**) 100 nm.

Different resolution FESEM images of CNTs-modified electrode are shown in Figure 2 which revealed that CNTs were successfully grown on graphite felt electrode. Figure 2a shows that CNTs were grown inside-out of fibrous structure of graphite felt. A uniform distribution of CNTs can be seen in Figure 2b, which also demonstrates the complete coverage of CNTs on graphite felt strands. Figure 2c gives the structural features of grown CNTs, long and thin CNTs are deposited on graphite felt.

### 3.2. Electrochemical Measurements

**Figure 3:**
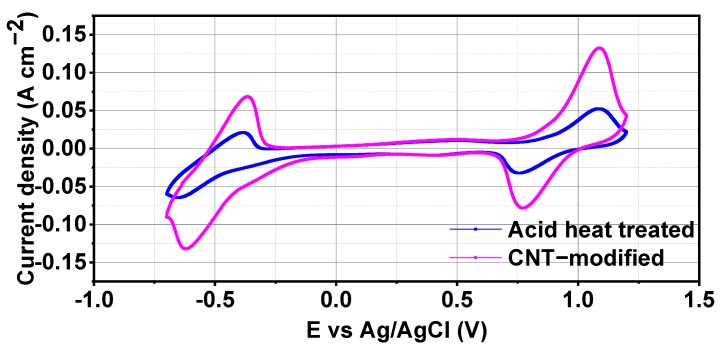
CV of CNTs-modified and acid heat treated electrodes in vanadium electrolyte.

CV curves of CNTs-modified electrode and acid heat treated electrode are shown in Figure 3. CV results showed two distinct peaks corresponding to V^3+^/V^2+^ (−1–0 V) and VO_2_^+/^VO^2+^ (0.5–1.5 V) vanadium redox pairs. Current responses of CNTs-modified electrode are significantly higher as compared to acid heat treated electrode because of presence of conducting CNTs. Similar kinds of current response behavior were observed in CNTs-modified electrode for both V^3+^/V^2+^ and VO_2_^+/^VO^2+^ redox pairs. Anodic (−I_pc_) and cathodic currents (I_pa_), −I_pc_(A)/I_pa_(A), anodic potential (E_pc_(V)), cathodic potential (E_pa_ (V)) and E (ΔV) of V^3+^/V^2+^ and VO_2_^+^/VO^2+^ for both electrodes are listed in Table 1 and Table 2, respectively.

In case of V^3+^/V^2+^ redox pair, anodic (−I_pc_) and cathodic currents (I_pa_) of CNTs-modified electrode were −0.132 and 0.068, respectively which were higher than −I_pc_ (−0.065) and I_pa_ (0.021) of acid heat treated electrode. Also, E_pa_ and E_pc_ for CNTs-modified electrode is less than acid heat treated electrode which means that electrochemical reactions are more feasible in CNTs-modified electrode. Similar results were reported by Li et al. [23] which revealed that CNTs presence decreased the reaction potential of the electrode thus ions can react more easily on electrode surface. A lower oxidation potential also indicates that VRFB will have better energy storage efficiency. −I_pa_(A)/I_pc_ (A) ratio and E (ΔV) signify the reaction kinetics of the electrode and electrolyte. −I_pa_(A)/I_pc_ (A) ratio suggests the reversibility of the reactions and E (ΔV) indicates the threshold energy required for a particular reaction [24]. CNTs-modified electrode had slightly low E (ΔV) and −I_pa_(A)/I_pc_ (A) shifted and became closer to 1 which means reaction kinetics of ions and reaction reversibility were improved with the presence of CNTs. Also, acid heat treated graphite felt electrode showed a low reaction reversibility for V^3+^/V^2+^ pair as compared to VO_2_^+/^VO^2+^ redox pair but in CNTs-modified electrode, both the reactions become prominent and reversible. This change in electrochemical behaviour of CNTs-modified electrode was because of conductivity of CNTs which act as electrocatalysts for the vanadium ions.

Similar kinds of responses were observed for VO_2_^+/^VO^2+^ redox pair, CNTs-modified electrode exhibited significantly higher −I_pc_, I_pa_ currents and a lower E (ΔV) as compared to acid treated electrode as reported in Table 2. All these findings from electrochemical study suggest the better electrochemical behaviour of CNTs-modified electrode than the acid heat treated electrode.

### 3.3. Battery Test

#### 3.3.1. Effect of Electrolyte Flow Rates

**Figure 4:**
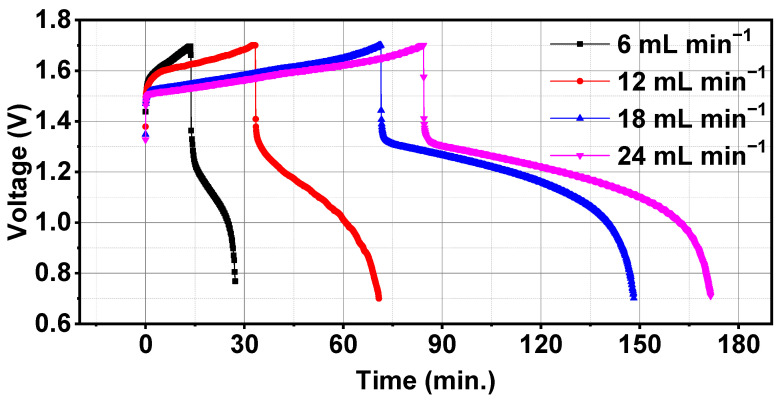
Comparison of voltage vs. time for various electrolyte flow rates with a constant current density of 40 mA cm^−2^.

Battery was tested at various electrolyte flow rates of 6, 12, 18, and 24 mL min^−1^ with a constant current density of 40 mA cm^−2^. Table 3 lists the charge-discharge capacity, charge-discharge energy, voltage efficiency, Coulombic efficiency and energy efficiency of the battery operating at various electrolyte flow rates. It can be observed from Table 3 that with little fluctuation of Coulombic efficiencies, charge-discharge capacities and efficiencies were increased with an increase in electrolyte flow rates. It was due to an increase in the supply of reactant species to the electrode and decreasing the mass transport resistance. Similar results have been reported by many groups [25,26,27] which showed that at low flow rates, electrolytes are not evenly distributed in VRFB and decrease the mass transfer coefficient, thus decreasing the capacities. Voltage time variation of first cycle for various electrolyte flow rates are shown in Figure 4. 24 mL min^−1^ flow rate showed the longest charge-discharge cycle time which was because of highest capacities among all the electrolyte flow rates. At 24 mL min^−1^, battery exhibited Coulombic efficiency and voltage efficiency of 91.45% and 74.90%, respectively, which was much higher than the Coulombic efficiency of 66.59% and voltage efficiency of 67.61% at 6 mL min^−1^ electrolyte flow rates. Battery offered 23.48% higher energy efficiency at a 24 mL min^−1^ electrolyte flow rate as compared to a 6 mL min^−1^ electrolyte flow rate. Charge-discharge capacities showed similar trends and increased from 0.18 to 1.63 Ah and from 0.12 to 1.49 Ah, respectively.

#### 3.3.2. Effect of CNTs-Modified Electrode

**Figure 5:**
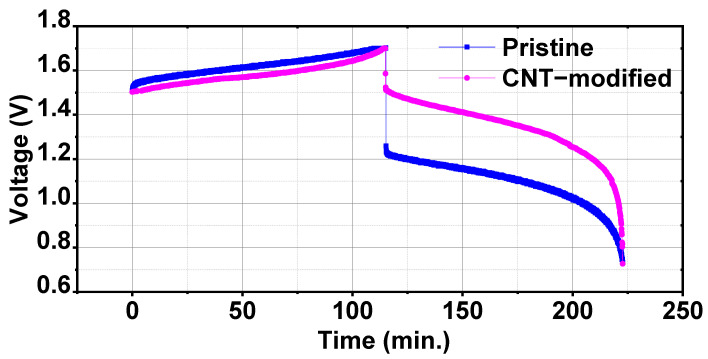
Comparison of voltage vs. time for pristine and CNTs-GF electrodes with a constant current density of 40 mA cm^−2^.

To study the effect of CNTs-modified electrodes, performance of the battery consisting of CNTs-modified electrodes was compared with the battery consisting of pristine graphite felt electrodes. Battery performances were recorded at a constant current density of 40 mA cm^−2^. Charge-discharge capacities were slightly decreased with CNTs-modified electrodes but discharge energy was more in CNTs-modified electrodes battery as compared to pristine electrodes battery. CNTs-modified electrodes battery offered a Coulombic efficiency of 96.30% and voltage efficiency of 79.33% which gave an energy efficiency of 76.39%. In case of pristine graphite felt electrodes, Coulombic efficiency was 94.47% and voltage efficiency was 65.08% which was equivalent to 61.48% energy efficiency. Energy efficiency of the CNTs-modified electrodes battery increased by 10% as compared to pristine electrodes battery. Enhanced performance of CNTs-modified electrodes battery was due to an increase in the conductivity of the electrodes because of CNTs presence. Electrochemical measurements showed that CNTs-modified electrodes have very good electrocatalytic properties toward both V^3+^/V^2+^ and VO_2_^+/^VO^2+^ redox pairs which gave an ease in oxidation-reduction reactions of electrolyte on electrode surface. This behaviour of electrode was similar to the other carbon and metal oxide based electrocatalysts for RFBs which also enhanced the reaction kinetics of V^3+^/V^2+^ and VO_2_^+/^VO^2+^ redox pairs in a similar way [28,29]. A comparison of voltage-time variation of the first charge-discharge cycle of CNTs-modified and pristine electrodes batteries is shown in Figure 5. CNTs-modified electrodes battery showed a quick charge-discharge as compared to pristine electrodes battery which was due to increased feasibility of the redox reactions. But overall charge and discharge energies increased from 3.20 Wh and 1.97 Wh for pristine electrode to 3.98 Wh and 3.06 Wh for CNTs-modified electrode, respectively. A comparison of performance parameters of two different electrodes batteries is reported in Table 4.

#### 3.3.3. Performance at Various Current Densities

**Figure 6:**
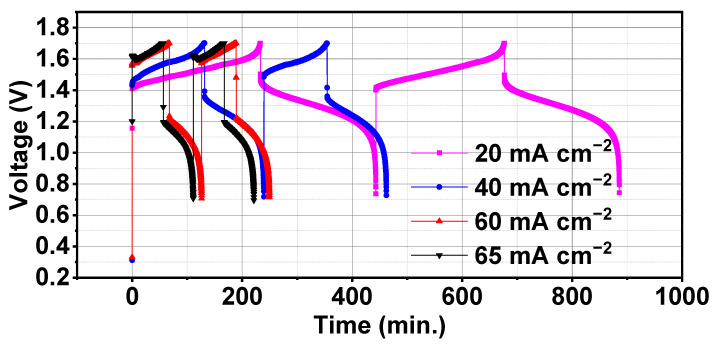
Comparison of voltage time variation of first two charge-discharge cycles of battery at 20, 40, 60, and 65 mA cm^−2^ current densities.

Effect of varying current densities was studied on a battery consisting of CNTs-modified electrodes. Current densities were varied at 20, 40, 60, and 65 mA cm^−2^ and resulting battery performance can be analysed from the data reported in Table 5. Voltage time variation of first two charge-discharge cycles for all current densities is given in Figure 6. It can be observed that charge-discharge time decreased with an increase in current density as longest and smallest charge-discharge cycles were recorded for 20 and 65 mA cm^−2^, respectively. Voltage efficiency drastically decreased with an increase in current density with a little fluctuation in Coulombic efficiency, overall energy efficiency of the battery decreased. This is because of an increase in the polarization losses at higher currents which lead to decreased energy efficiency [15,30]. This can also be observed from charge and discharge capacities trends which values were decreasing with increasing current densities. Battery performance was consistent with reported data on VRFB which suggested that capacity utilization decrease results in efficiency losses. Voltage time graphs also showed that increasing current density decreases the charge-discharge time because appropriate state of charge (SOC) limit does not support high currents [25,31]. Charge-discharge capacities showed a small increase when current density increased from 20 to 40 mA cm^−2^ but that only increased charge energy overall discharge energy was decreasing for each increased value of current density. Energy efficiency obtained at 20 mA cm^−2^ was 75.79% which decreased to 63.08% at 65 mA cm^−2^. A similar trend was observed for discharge energy which decreased from 2.24 Wh to 1.54 Wh with changing current density from 20 to 40 mA cm^−2^. Performance can be improved by increasing the electrolyte flow rates for matching the SOC at higher current densities but that will result in power losses which again degrade the system performance. Thus, an optimum current efficiency with an optimum electrolyte flow rate is acceptable for good battery performance.

#### 3.3.4. Effect of Removing Current Collector Plates

**Figure 7:**
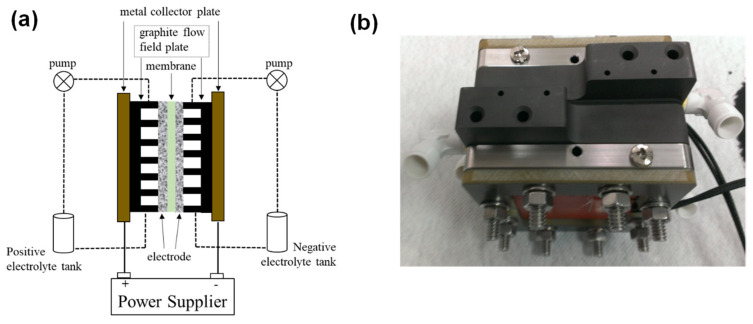
(**a**) Schematic of VRFB consisting of metal collector plates (**b**) a photograph of battery without current collector plates.

Current collector plates are an important part of battery assembly and these can significantly affect the resistance of the battery. It was reported that current collectors of different types of materials can result in different RFB performances [32]. Wang et al. [33] designed a pluggable current collector for in-operando current measurements and analysed the factors of performance degradation in VRFBs. It was concluded that uneven current distribution was due to a shortage of reactant species in the broader region of electrodes. A theoretical study on large scale Li-ion batteries revealed that current collector plates contribute 10% of total battery resistance which was considerably large as compared to bulk resistance which contributed 3% of total battery resistance [34]. Thus, current collector plates resistance can significantly reduce battery performance. Here, current collector plates were removed from the battery assembly and tester wires were directly connected to the graphite plates as shown in Figure 7. Battery performance without current collector plates was compared with the battery consisting of current collector plates. Performance comparison of two types of configurations can be done by comparing performance parameters listed in Table 6. Discharge capacity with current collector plates was 1.56 Ah which increased to 1.80 Ah and energy efficiency was also 10% higher for battery without current collector plates. Energy efficiency with current collector plates was 60.25%, which increased to 70.30% for battery without current collector plates. This enhancement in the battery performance without current collector plates configuration was due to absence of the resistance caused by current collector plates.

**Figure 8:**
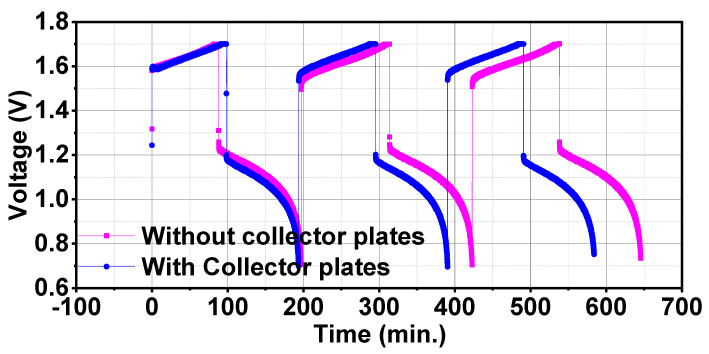
Comparison of voltage-time variation of charge-discharge cycle for batteries with and without current collector plates.

As charge-discharge energy capacities increase in without current collector plates battery configuration, so voltage-time variation showed a longer charge-discharge cycle time as shown in Figure 8. This signifies that collector plates contributed a large resistance to the battery total resistance thus battery performance can be improved by developing new designs and using less resistive materials for making of current collector plates.

## 4. Conclusions

In this study, a CVD-grown CNTs-modified electrode was prepared and various aspects of VRFBs were examined. CNTs-modified electrode electrochemical performance was compared with acid heat treated graphite felt electrode. CNTs-modified electrode gave improved reaction kinetics of vanadium redox pairs due to enhanced conductivity of the electrode. VRFB performance was analysed, at various electrolyte flow rates, at different current densities, and with and without collector plates battery configurations. Battery consisting of CNTs-modified electrodes showed better performance as compared to pristine electrodes. Energy efficiency obtained with CNTs-modified electrodes battery was 76.39% at 40 mA cm^−2^ which was 15% higher than the pristine electrodes. VRFB performance improved with increasing electrolyte flow rates as it increased the energy capacities. At higher current densities, battery performance degraded due to an increase in the polarization losses at higher currents. Battery without current collector plates showed considerable efficiency improvement due to a decrease in battery resistance. Battery consisting of current collector plates showed 60.25% energy efficiency which increased to 62.93% without current collector battery configuration.

## Figures and Tables

**Table 1:** −I_pc_, I_pa,_ −I_pc_(A)/I_pa_ (A), E_pa_ (V), E_pc_(V) and E (ΔV) of V^3+^/V^2+^ for CNTs-modified electrode and acid heat treated electrode.

Electrode Type	−I_pc_(A cm^−2^)	I_pa_ (A)	−I_pc_(A)/I_pa_(A)	E_pa_ (V)	E_pc_(V)	E (ΔV)
Acid-heat treatment	−0.065	0.021	0.323	−0.393	−0.652	0.259
CNTs-modified	−0.132	0.068	0.515	−0.361	−0.615	0.254

**Table 2:** −I_pc_, I_pa,_ −I_pc_(A)/I_pa_(A), E_pa_ (V), E_pc_(V) and E (ΔV) of VO_2_^+/^VO^2+^ for CNTs-modified electrode and acid heat treated electrode.

Electrode Type	−I_pc_(A)	I_pa_(A)	−I_pc_(A)/I_pa_(A)	E_pa_(V)	E_pc_(V)	E (ΔV)
Acid-heat treatment	−0.032	0.052	1.625	1.097	0.756	0.341
CNTs-modified	−0.078	0.132	1.692	1.088	0.768	0.320

**Table 3:** Effect of electrolyte flow rates on the various performance parameters of battery.

Flow Rate (mL min^−1^)	Charge Capacity (Ah)	Discharge Capacity (Ah)	Charge Energy (Wh)	Discharge Energy (Wh)	Coulombic Efficiency (%)	Voltage Efficiency (%)	Energy Efficiency (%)
6	0.18	0.17	0.29	0.19	97.88	67.61	66.18
12	0.72	0.69	1.14	0.76	96.40	68.50	66.03
18	1.33	1.22	2.11	1.41	92.05	72.31	66.56
24	1.63	1.57	2.51	1.81	96.17	74.90	72.04

**Table 4:** Performance comparison of CNTs-modified electrodes battery with pristine electrodes battery.

State	Charge Capacity (Ah)	Discharge Capacity (Ah)	Charge Energy (Wh)	Discharge Energy (Wh)	Coulombic Efficiency (%)	Voltage Efficiency (%)	Energy Efficiency (%)
pristine	1.95	1.85	3.20	1.97	94.47	65.08	61.48
CNTs-modified	2.01	1.95	3.98	3.06	96.30	79.33	76.39

**Table 5:** Performance parameters of battery at various current densities of 20, 40, 60, and 65 mA cm^−2^.

Current Density (mA cm^−2^)	Charge Capacity (Ah)	Discharge Capacity (Ah)	Charge Energy (Wh)	Discharge Energy (Wh)	Coulombic Efficiency (%)	Voltage Efficiency (%)	Energy Efficiency (%)
20	1.94	1.75	2.96	2.24	90.10	84.11	75.79
40	2.19	1.80	3.44	2.17	83.20	76.63	63.07
60	1.69	1.46	2.76	1.60	86.27	67.27	58.04
65	1.69	1.43	2.43	1.54	96.36	65.47	63.08

**Table 6:** Performance comparison of with and without current collector plates configurations of batteries.

State	Charge Capacity (Ah)	Discharge Capacity (Ah)	Charge Energy (Wh)	Discharge Energy (Wh)	Coulombic Efficiency (%)	Voltage Efficiency (%)	Energy Efficiency (%)
With current collector plates	1.64	1.56	2.71	1.65	94.03	64.08	60.25
without current collector plates	1.93	1.80	4.17	2.62	93.43	67.36	62.93

## Data Availability

The data presented in this study are available on request from the corresponding author.
